# Use of health care services by people with substance use disorders in Belgium: a register-based cohort study

**DOI:** 10.1186/s13690-021-00620-5

**Published:** 2021-06-23

**Authors:** Luk Van Baelen, Els Plettinckx, Jérôme Antoine, Karin De Ridder, Brecht Devleesschauwer, Lies Gremeaux

**Affiliations:** 1 Sciensano, Department of Epdemiology and public health, Rue Juliette Wytsmanstraat, 14, 1050 Brussels, Belgium; 2 Department of Veterinary Public Health and Food Safety, Ghent University, Merelbeke, Belgium

**Keywords:** Substance Use Disorders, Health care services, Health care service providers, Belgium, Cohort study, Epidemiology

## Abstract

**Background:**

The objective of the study was to describe the frequencies of health-care utilization by people with substance use disorder (SUD), including contacts with general practitioners (GP), psychiatrists, emergency departments (ED) and hospital admissions and to compare this frequency to the general population.

**Methods:**

Data from the national register of people who were in treatment for SUD between 2011 and 2014 was linked to health care data from the Belgian health insurance (N = 30,905). Four comparators were matched on age, sex and place of residence to each subject in treatment for SUD (N = 123,620). Cases were further divided in five mutually exclusive categories based on the main SUD (opiates, crack/cocaine, stimulants, cannabis and alcohol). We calculated the average number of contacts with GP, psychiatrists and ED, and hospital admissions per person over a ten year period (2008–2017), computed descriptive statistics for each of the SUD and used negative binomial regression models to compare cases and comparators.

**Results:**

Over the ten-year period, people in treatment for SUD overall had on average 60 GP contacts, 3.9 psychiatrist contacts, 7.8 visits to the ED, and 16 hospital admissions. Rate ratios, comparing cases and corresponding comparators, showed that people in treatment for SUD had on average 1.9 more contacts with a GP (95 % CI 1.9-2.0), 7.4 more contacts with a psychiatrist (95 % CI 7.0-7.7), 4.2 more ED visits (95 % CI 4.2–4.3), and 6.4 more hospital admissions (95 % CI 6.3–6.5).

**Conclusions:**

The use of health services for people with SUD is between almost two (GP) and seven times (psychiatrist) higher than for comparators. People in treatment for alcohol use disorders use health care services more frequently than people in treatment for other SUD. The use of health services remained stable in the five years before and after the moment people with SUD entered into treatment for SUD. The higher use of primary health care services by people with SUD might indicate that they have higher health care needs than comparators.

## Background

People with substance use disorders (SUD) are known to have poor health outcomes and increased risk of premature death. In Belgium, previous research found that being in treatment for SUD increased all-cause mortality risk nearly elevenfold for users of illicit drugs, and sevenfold for users of licit drugs [1]. In 2017 in Belgium, alcohol was estimated to account for 5.3 % of all deaths and 6.1 % of the disability-adjusted life-years (DALYs) [2]. In the same year (2017), illicit drugs were estimated to contribute to 0.25 % of all deaths and to 0.81 % of the DALYs. People with SUD have a higher risk of contracting cancers, cardiovascular, respiratory and liver disorders [3, 4] or infectious diseases such as tuberculosis [5], hepatitis C [6] or diseases due to the human immunodeficiency virus (HIV) [7], and also more oral health problems [8].

Notwithstanding these high needs, people with SUD face many obstacles when in need for general health care treatment: lack of financial means, lack of knowledge about the availability of help, lack of trust in treatment, and stigmatizing attitudes among health professionals [9–12]. For example, one of the reasons for the low number of people with SUD treated for Hepatitis C Virus (HCV) is some clinicians considering them as difficult to treat because of existing social and psychological barriers and concerns about reinfection [13–15]. However, recent studies in Belgium [16, 17] have shown that there is no significant difference in outcome between HCV treatment of people with SUD and the general population. Several other national and international studies have reported that treatment barriers not only exist in the case of infectious diseases such as hepatitis C or HIV [6, 7] but also for general health care [18, 19], hospital care [18, 20], dental health care [8], palliative care [21], and preventive measures such as contraception [22] or for access to pharmacies [18]. Although scientific literature on these obstacles is lacking for people with SUD in Belgium, there is no reason to believe that they face less barriers for these health problems than people in other parts of the world.

Because of these obstacles, previous studies have concluded that people with SUD heavily rely on emergency departments for general healthcare problems [23, 24]. A meta-analysis in 2019 revealed that people with SUD have on average 4.8 times more episodes in emergency departments than the general population [25]. The same review also identified several gaps in the evidence such as the fact that little is known about the health care use of people with cannabis use disorders, MDMA or amphetamine use disorders, powder cocaine use disorders, as well as the lack of knowledge about the use of primary health care by people with SUD [25]. It remains unclear whether the higher use of ED visits and hospitalizations are indeed the result of postponed care due to structural, financial and social obstacles, or if the needs for PWUD are generally much higher, which could also result in more frequent use of primary health care.

To address these gaps, the primary objective of this study is to describe the frequencies of health-care utilization by people with SUD, more specifically contacts with general practitioners (GP), psychiatrists, emergency departments (ED) and hospital admissions, for people with alcohol use disorders, cannabis use disorders, disorders related to the use of stimulants other than crack/cocaine, cocaine use disorders and opiate use disorders. The second objective is to compare the frequency of health-care utilization of these people to the general population in Belgium.

## Methods

Data for the current study was generated through the linkage and matching of two existing Belgian national health and population registers: (1) the Belgian Treatment Demand Indicator database (TDI) with information on socio-demographic variables and substances for which treatment was sought at the start of the treatment episode for people in treatment for SUD, covering almost all specialized drug treatment centers and by around one third of the general or psychiatric hospitals [26], and (2) the InterMutualistic Agency database (IMA, [27]) with data on reimbursed health care services, gathered through the seven Belgian health insurance agencies. The data that was used from this database consists of contacts with general practitioners, psychiatrists, ED and admissions to the hospital, and spanned a period between 1 January 2008 and 31 December 2017. The full IMA-database covers 99 % of the people living in Belgium, as health care insurance is mandatory [28].

The Belgian National Identification Number (NIN) was used to link both databases. All patients registered in TDI with a NIN who started a treatment for SUD between 2011 and 2014 and who could be identified in the IMA database were considered eligible subjects for this study (n = 30,905). For those with multiple episodes the first treatment episode in TDI was selected, in line with the TDI-IMA protocol [28]. Cases were further divided in five mutually exclusive categories: people in treatment for opiate use disorders (some of which were also in treatment for other substances), people in treatment for crack/cocaine use disorders who were not in treatment for opiates, people in treatment for stimulant use disorders (mainly MDMA and amphetamines) who were not in treatment for opiates or crack/cocaine, people in treatment for cannabis use disorders with or without alcohol use disorders who were not in treatment for opiates, crack/cocaine or stimulants, and people in treatment for only alcohol use disorders.

Furthermore, there were 3,198 people in treatment for other substances, who were excluded from the analysis, which gives a total of 27,707 cases.

A group of comparators who had not been in treatment for SUD between 2011 and 2014 was selected from the general population through the IMA database [28]. Four comparators were matched on age, sex and municipality of residence to each case in treatment for SUD. Sex and age were considered to be basic matching variables. The potential confounding of municipality of residence is related to regional differences in health care regulation, health care seeking and access to specialized medical health care for SUD as well as other differences that might be present, for instance socio-economic status of the patients by region.

For people with SUD as a whole as well as for each of the five patient categories and the corresponding comparators and for men and women separately, we calculated the median and average number of contacts with primary care physicians, psychiatrists and ED, and hospital admissions per person over the ten year period. We computed descriptive statistics for each of these groups and used negative binomial regression models to compare cases and comparators. This analysis models the log of the expected count (i.e., number of visits) as a linear function of cases versus comparators. The coefficients obtained were exponentiated to get rate ratios (with corresponding 95 % confidence intervals). For the graphical representation of the average use of each outcome variable data were recalibrated towards the day people with SUD entered into treatment. Graphs represent the results of the outcome variable five years before and five years after this day. In this way, they illustrate the evolution over time per month for each category of patients and corresponding comparators.

Data analysis was done using SAS software version 9.3 (SAS Institute Inc., Cary, NC). The reporting of this study conforms to the STROBE guidelines [29].

## Results

As shown in Table 1, almost one in three cases was in treatment for alcohol use disorders only, one in five was in treatment for cannabis or opiate use disorders, whereas 14 % was in treatment for cocaine use disorders and 8.6 % for stimulant use disorders.

**Table 1:** Descriptive statistics people in treatment for substance use disorder by main product in Belgium (2011-2014)

			**opiates**	**crack/cocaine**	**stimulants**	**cannabis**	**alcohol**
Sex (N missing = 9)	Men	N	4502	3420	1998	4924	6207
		%	79.1%	80.9%	74.8%	85.3%	66.5%
	Women	N	1188	807	675	852	3125
		%	20.9%	19.1%	25.3%	14.8%	33.5%
Age (N missing = 2365)	<20	N	68	177	308	1363	26
		%	1.2%	4.4%	12.3%	25.8%	0.3%
	20-29	N	1568	1737	1038	2236	388
		%	28.5%	43.4%	41.4%	42.3%	4.7%
	30-39	N	2153	1516	839	1125	1322
		%	39.2%	37.9%	33.4%	21.3%	16.0%
	40-49	N	1380	480	260	435	2690
		%	25.1%	12.0%	10.4%	8.2%	32.6%
	50-59	N	299	83	59	114	2686
		%	5.4%	2.1%	2.4%	2.2%	32.5%
	>60	N	25	10	6	11	1147
		%	0.5%	0.2%	0.2%	0.2%	13.9%
Region (N missing = 0)	Flanders	N	3096	3345	2604	4785	6298
		%	54.4%	79.1%	97.4%	82.8%	67.5%
	Wallonia	N	1815	602	43	768	2591
		%	31.9%	14.2%	1.6%	13.3%	27.8%
	Brussels	N	780	280	26	227	447
		%	13.7%	6.6%	1.0%	3.9%	4.8%
In treatment before (N missing = 1768)	No	N	965	1969	1215	3547	3295
		%	19.3%	49.1%	48.6%	63.4%	36.6%
	Yes	N	4035	2042	1283	2045	5709
		%	80.7%	50.9%	51.4%	36.6%	63.4%
Type treatment setting (N missing = 0)	Inpatient	N	2341	1675	761	1506	7918
		%	41.1%	39.6%	28.5%	26.1%	84.8%
	Outpatient	N	3350	2552	1912	4274	1418
		%	58.9%	60.4%	71.5%	73.9%	15.2%
Total		N	5691	4227	2673	5780	9336
		%	18.4%	13.7%	8.6%	18.7%	30.2%

People in treatment for alcohol use disorders were older, were more likely to have been in treatment for SUD before and relied mainly on inpatient services. People in treatment for disorders related to street drugs were younger and most often supported by outpatient services.

Over the ten-year period 2008–2017, people in treatment for SUD overall had on average 60 GP contacts, 3.9 psychiatrist contacts, 7.8 visits to the ED, and 16 hospital admissions (Table 2).

**Table 2:** Median and mean number of contacts/episodes with health provider/service (standard deviation (sd)) per 10 person-years for people in treatment for substance use disorders between 2011 and 2014 and their comparators in Belgium, overall, by substance and by sex (2008-2017)

			**Contacts with GP**^a^			**Contacts with psychiatrist**			**Contacts with ED**^a^			**Hospital admissions**		
		n	median	mean	sd	median	mean	sd	median	mean	sd	median	mean	sd
overall	control	123620	23	30.8	33.3	0	0.5	1.2	1	1.9	2.9	1	2.5	9.4
	case	30905	43	59.9	68.4	0	3.9	10.8	5	7.8	11.9	6	16.0	30.8
opiates	control	22764	19	26.1	29.4	0	0.6	4.5	1	2.1	3.3	1	2.2	9.7
	case	5691	44	68.5	82.7	0	3.9	14.1	5	7.9	10.8	5	10.1	21.4
cocaine/crack	control	16908	20	26.7	27.9	0	0.4	3.7	1	1.9	2.8	1	2.1	10.0
	case	4227	36	47.0	49.1	0	2.7	7.8	5	7.0	8.4	4	10.6	23.1
stimulants	control	10692	23	29.1	27.5	0	0.4	3.1	1	1.8	2.6	1	2.2	8.7
	case	2673	40	53.3	55.7	0	2.5	8.0	4	5.8	6.5	4	11.9	25.0
cannabis	control	23120	19	24.8	25.2	0	0.4	2.9	1	2.0	2.9	1	1.8	6.7
	case	5780	31	39.6	43.1	0	2.1	6.5	3	5.6	8.3	3	8.9	21.9
alcohol	control	37344	29	38.0	39.3	0	0.7	5.1	1	1.6	2.7	1	3.2	10.4
	case	9336	51	64.7	62.7	1	5.0	11.2	6	9.1	14.2	12	24.2	38.4
women	control	32268	33	41.6	40.6	0	0.7	4.4	1	1.7	3.0	2	3.4	9.5
	case	8067	62	81.9	84.1	1	4.9	11.8	6	8.6	12.7	11	22.5	13.3
men	control	91316	20	26.9	29.3	0	0.5	4.1	1	1.9	2.9	1	2.2	9.4
	case	22829	37	52.1	60.1	0	3.5	10.4	5	7.6	11.6	5	13.7	27.8

^a^*GP* general practitioner, *ED* emergency department

Rate ratios, comparing cases and corresponding comparators, showed that people in treatment for SUD had on average 1.9 more contacts with a GP (95 % CI 1.9-2.0), 7.4 more contacts with a psychiatrist (95 % CI 7.0-7.7), 4.2 more ED visits (95 % CI 4.2–4.3), and 6.4 more hospital admissions (95 % CI 6.3–6.5) (Table 3).

**Table 3:** Rate ratios (RR) (with standard error (SE) and 95 % confidence interval (CI)) of use of health provider/service for people in treatment for substance use disorders between 2011 and 2014 versus comparators in Belgium, overall, by substance and by sex (2008–2017)

	Contacts with GP^a^			Contacts with psychiatrist			Contacts with ED^a^			Hospital admissions		
	*RR*	*SE*	*95 % CI*	*RR*	*SE*	*95 % CI*	*RR*	*SE*	*95 % CI*	*RR*	*SE*	*95 % CI*
Overall	1.9	0.01	1.9-2.0	7.4	0.17	7.0-7.7	4.2	0.03	4.2–4.3	6.4	0.06	6.3–6.5
Opiates	2.6	0.04	2.5–2.7	6.7	0.40	5.9–7.5	3.7	0.06	3.5–3.8	4.6	0.10	4.4–4.8
Cocaine	1.8	0.03	1.7–1.8	6.5	0.40	5.7–7.4	3.6	0.07	3.5–3.8	5.0	0.12	4.8–5.3
Stimulants	1.8	0.03	1.8–1.9	7.0	0.61	5.9–8.3	3.3	0.08	3.1–3.5	5.3	0.16	5.0-5.6
Cannabis	1.6	0.02	1.6–1.6	5.6	0.30	4.9–6.3	2.8	0.05	2.7–2.8	4.9	0.10	4.7–5.1
Alcohol	1.7	0.02	1.7–1.7	7.7	0.30	7.1–8.3	5.7	0.05	5.6–5.9	7.6	0.11	7.3–7.8
Women (vs. men)	1.6	0.02	1.5–1.6	1.4	0.04	1.3–1.5	1.1	0.02	1.1–1.2	1.7	0.03	1.6–1.7

^a^*GP *general practitioner, *ED *emergency department

Rate ratios per SUD category ranged for GP contacts between 1.6 (cannabis, 95 %CI 1.6–1.6) and 2.6 (opiates, 95 %CI 2.5–2.7), for psychiatrist contacts between 5.6 (cannabis, 95 %CI 4.9–6.3) and 7.7 (alcohol, 95 %CI 7.1–8.3), for contacts with ED between 2.8 (cannabis, 95 %CI 2.7–2.8) and 5.7 (alcohol, 95 %CI 5.6–5.9), and for hospital admissions between 4.6 (opiates, 95 %CI 4.4–4.8) and 7.6 (alcohol, 95 %CI 7.3–7.8).

Women with SUD were using health care services more than men, with rate ratios ranging between 1.1 (ED, 95 %CI 1.1–1.1) and 1.7 (hospital admissions, 95 % CI 1.6–1.7).

**Fig. 1:**
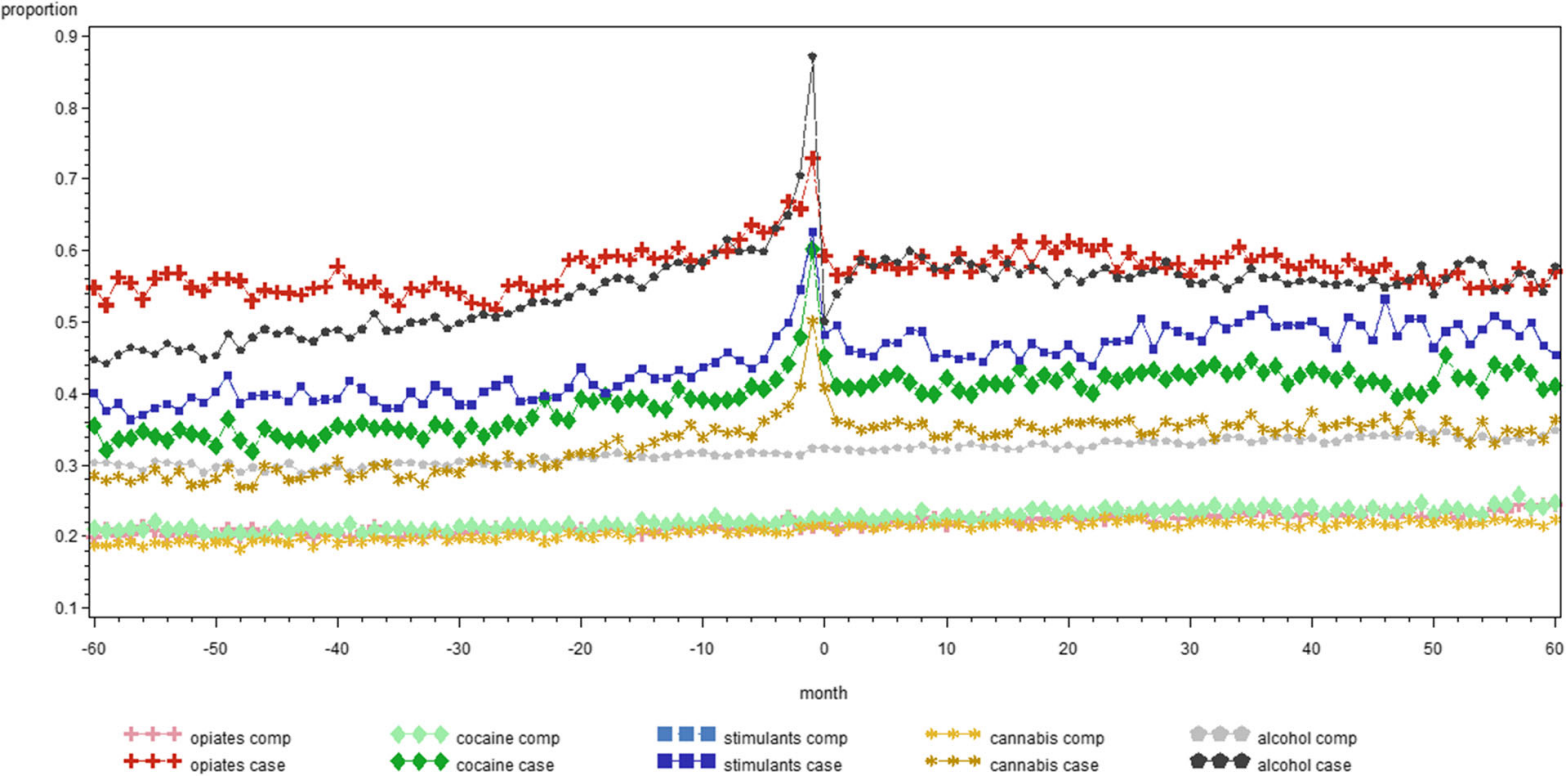
Monthly proportion of people in treatment for substance use disorders between 2011 and 2014 and comparators in contact with general practitioner in the five years before and after the day of start treatment, by substance in Belgium (2008-2017)

**Fig. 2:**
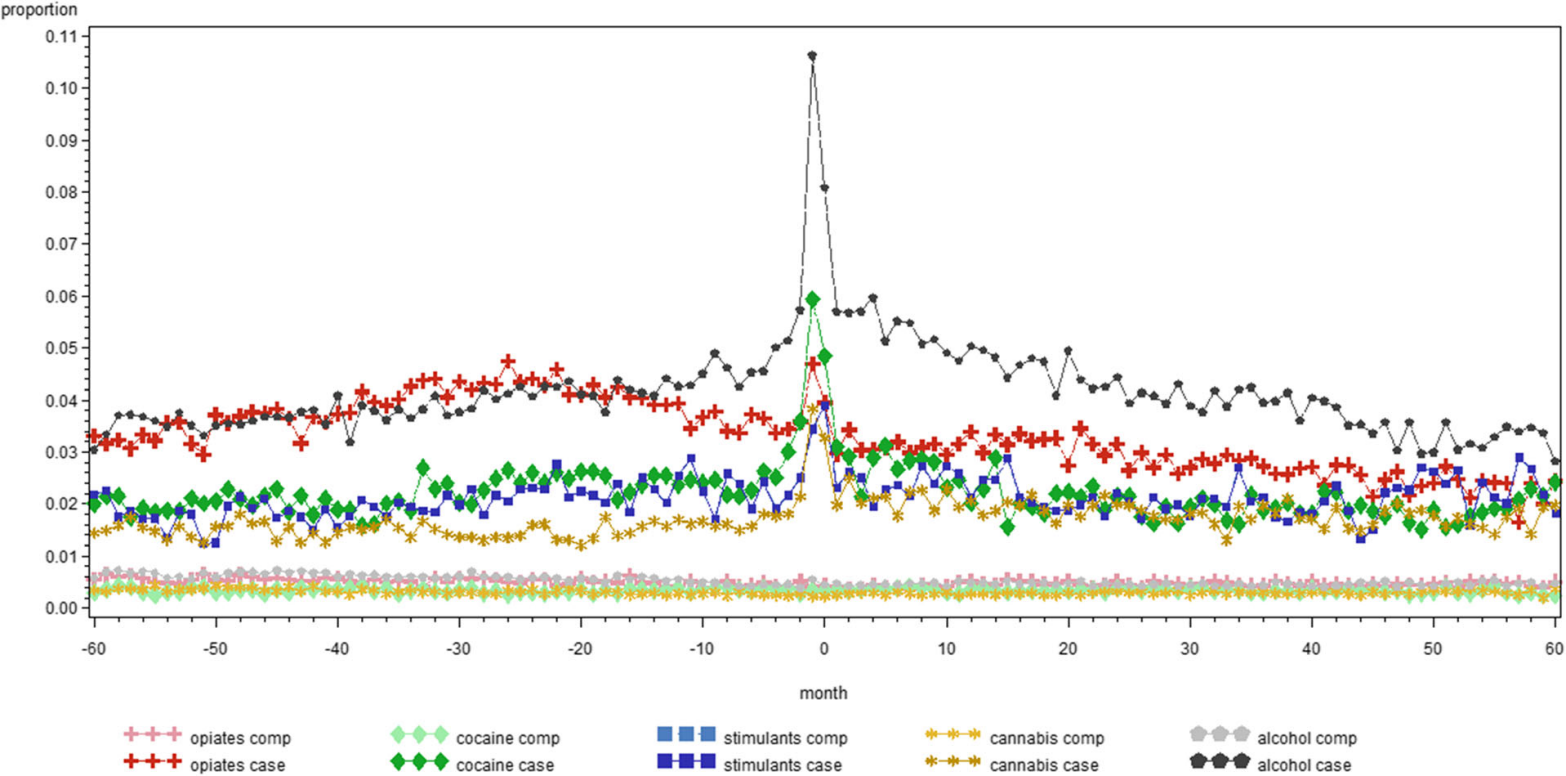
Monthly proportion of people in treatment for substance use disorders between 2011 and 2014 and comparators in contact with a psychiatrist in the five years before and after the day of start treatment, by substance in Belgium (2008-2017)

**Fig. 3:**
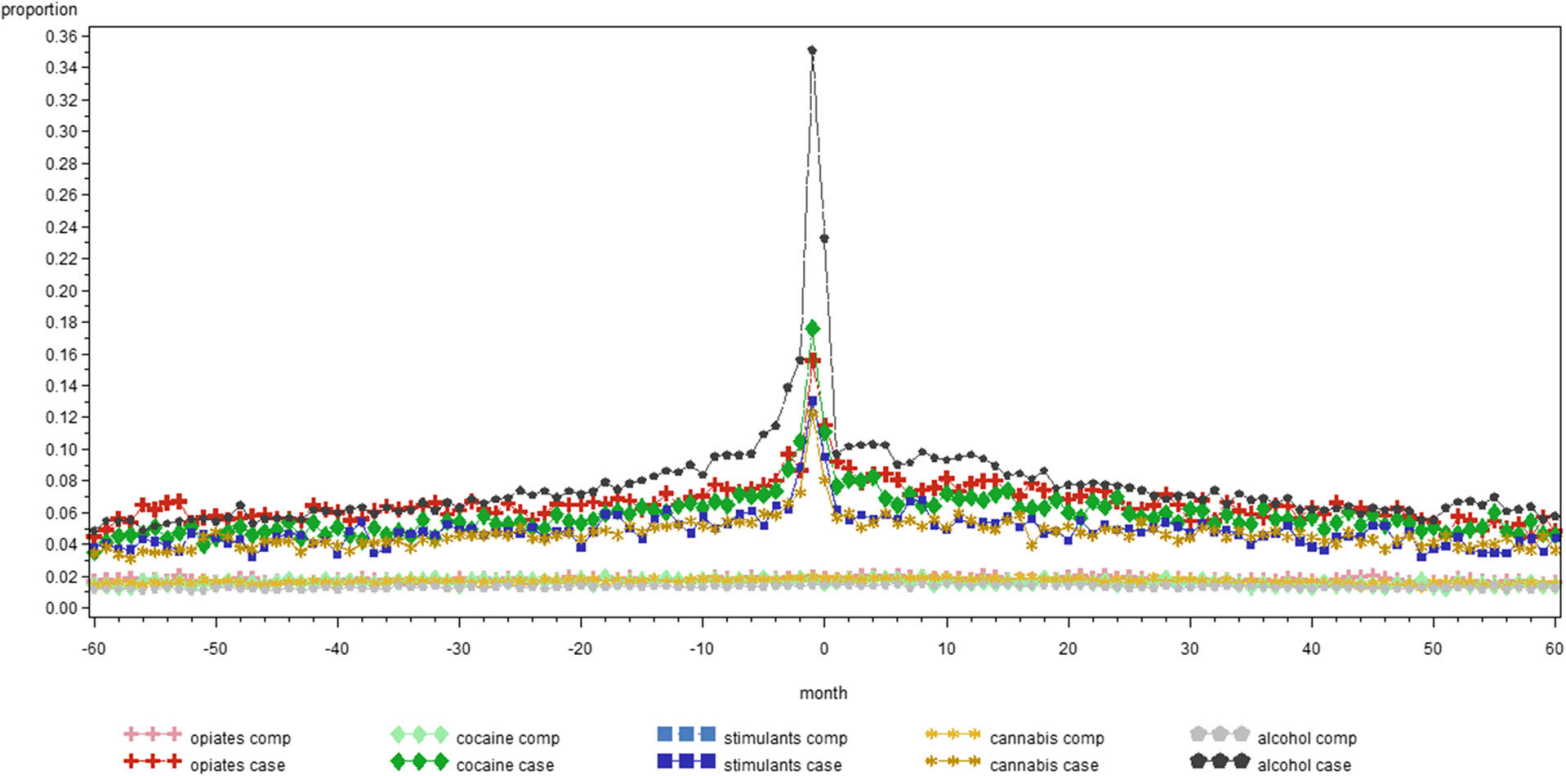
Monthly proportion of people in treatment for substance use disorders between 2011 and 2014 and comparators in contact with emergency department in the five years before and after the day of start treatment, by substance in Belgium (2008-2017)

**Fig. 4:**
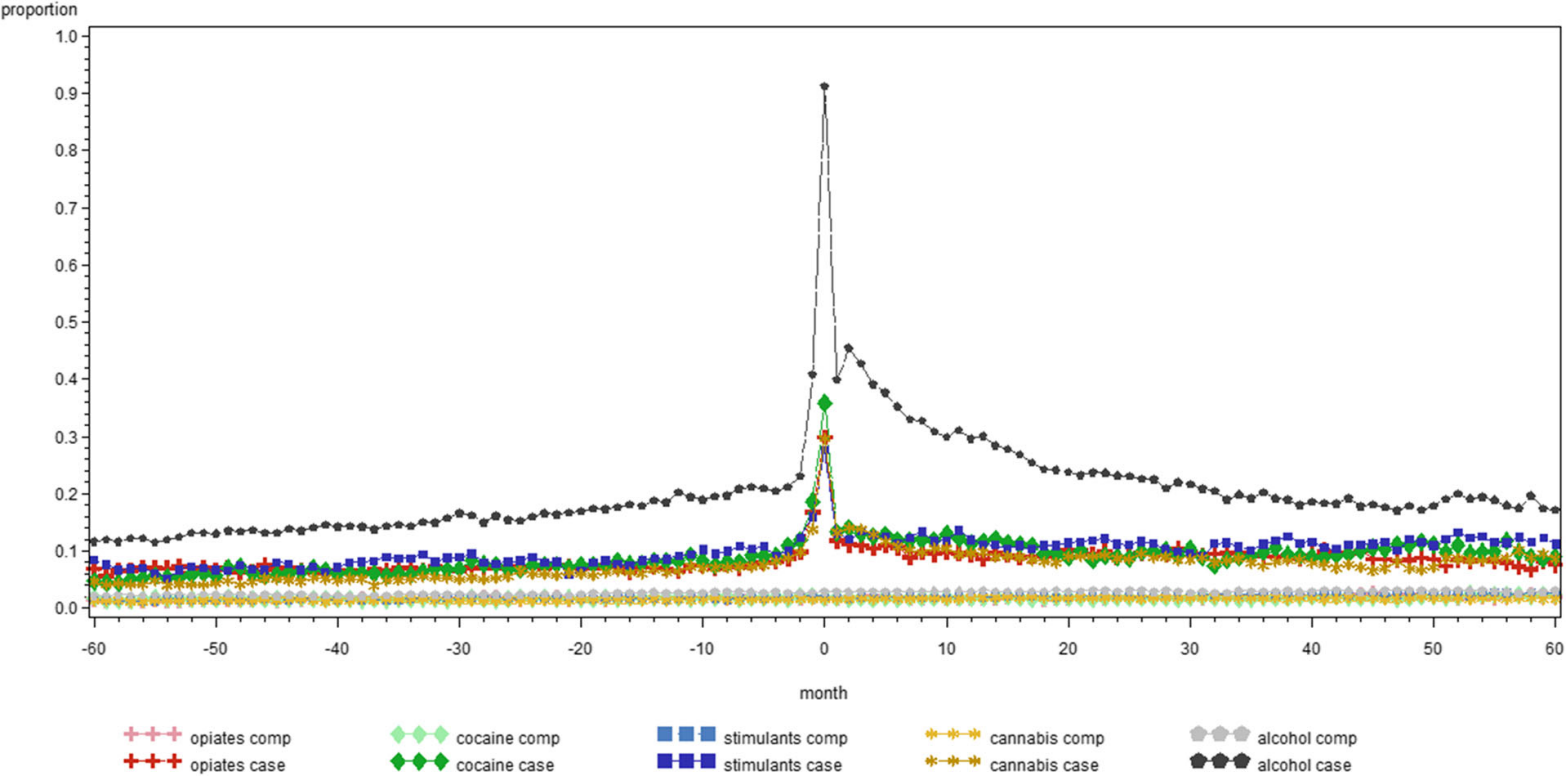
Monthly proportion of people in treatment for substance used disorders between 2011 and 2014 and comparators with hospital admissions in the five years before and after the day of start treatment, by substance in Belgium (2008-2017)

Figure 1 illustrates the monthly proportion of people in treatment for SUD in contact with GP for each category of SUD and their comparators. It reflects the data of Table 2 as monthly averages, e.g. people with alcohol use disorders have over the 120 months period on average 65 contacts with a GP, which give an average of approximately 0.5 contacts per month per person. Figures 2, 3 and 4 give a similar representation of the proportion of people in treatment for SUD and comparators in contact with psychiatrists, the ED and by hospital admissions, respectively.

## Discussion

Use of health care services by people with SUD is high. Our results for ED and hospitalizations episodes are in line with the results of previous studies [25]. At the same time, our study gives a better understanding of the number of contacts with general practitioners by people with SUD. People in treatment for SUD have between 1.6 and 1.9 times more contacts with a GP than their comparators, except for people in treatment for opiate use disorders who have on average 2.6 times more contacts with their GP than their respective comparators. The reason for this higher frequency might be that many of them are on substitution treatment for which they have to consult a GP. For all other considered health service providers, people with alcohol use disorders had more frequent contacts than people with disorders related to street drugs and the comparators. This last observation is in contrast to findings from a previous study where people with alcohol use disorders were found to use less health care services than the general population [30].

Overall, people with SUD had a higher use of primary care than acute health care, which is in line with findings from the few number of studies who reported on this [31, 32]. Compared to the comparators, the differences in use of health services between people in treatment for disorders related to cannabis, stimulants and crack/cocaine are minimal for contacts with the GP, and not significant for hospitalizations and contacts with psychiatrists. Only people in treatment for cannabis use disorders have significantly less contacts with the ED than the comparators compared to people with disorders related to stimulants and crack/cocaine.

Interestingly, as shown by the Figs. 1, 2, 3 and 4, there is little difference in the number of contacts with the specific health service providers over time within each category of SUD. Indeed, the use of health services remains quite stable in the five years before and after the moment people with SUD entered into treatment. Of course, this does not provide insights in individual variation over time, but it remains interesting to see that this level of health care use was that stable. We could not find other studies to support or contradict this finding.

One reason for the high use of general health services might be that the data is based on the first episode in TDI between 2011 and 2014, with between 36.6 % (cannabis) and 80.7 % (opiates) of the cases who had been already in treatment for SUD before, meaning that they might have had already serious substance use related and general health care problems before the day on which they were registered in TDI. We also acknowledge the fact that the proportion of people with hospital admissions in the months after start of treatment for alcohol cases (Fig. 4) shows a slower decline than for other substance categories, although at this time we cannot provide a good explanation for this.

A previous study concluded that the higher use of health care services by PWUD, compared to comparators from the general population, reflects the greater need for treatment, but that it does not necessarily represent good health-care access and that this might reflect a pattern where primary and preventative health care is poor and PWUD rely mainly on unplanned health care [25]. The current study gives a more nuanced picture. Indeed, PWUD make more use of ED and are more admitted to hospitals, but also the use of GPs is significantly higher than for comparators. This higher use of primary health care could underline the presence of high needs, as does the substantial higher use of psychiatric care. At the same time, the current study cannot conclude that access for PWUD is sufficient or that existing care services answer the needs of PWUD, since we only analyzed the use of health care and not outcome of care. For instance, postponing to seek and receive health care is likely to increase the risk of developing complications [33]. Even if PWUD attend more often than comparators a GP, it might still be that they wait much longer to seek help, which might result in more intense use of health care services.

The current study has several limitations, some of which have already been mentioned in previous articles [1, 6, 28]. Three out of four episodes in TDI are registered with a NIN. This means that for one quarter of all episodes it was impossible to identify the person with SUD and to extract their data from the IMA-database. People who are registered without NIN are known to be more in treatment in a psychiatric unit of a general hospital, in treatment for opiates, in treatment in Wallonia and to be non-Belgian [34], but as shown by a previous study, there is little evidence that patient characteristics have a major impact on the registration with a NIN [34].

Moreover, outreach services and harm reduction initiatives are not covered by TDI, nor are GPs who are considered to play a major role in detecting and managing SUD directly [26]. This means that people with SUD who are only treated by GPs are not included in the database. However, for all people registered in TDI, data about their consultations to GPs are registered in IMA. As a result the exact denominator of all people who are in treatment for SUD in Belgium is unknown, but the proportion of them who are registered in TDI can be understood as the large majority. Further research will look into this denominator. Because health insurance is mandatory in Belgium, for almost all people who are registered in TDI with a NIN, data are available in IMA. As such, the reported results give a reliable understanding of the use of services for general healthcare problems by people with SUD in Belgium who are registered in TDI with a NIN.

Finally, the use of register data for a vast and diversified group of people in treatment for SUD allows a better understanding of the number of contacts with health care service providers. However, the relatively limited evolution over time, and the fact that almost 60 % of the cases had been in specialized treatment for SUD before, supports the idea that the development of SUD is a long-term process. Further research should thus take a longer time period into consideration to describe earlier phases in the use of general health care services leading to specialized treatment of SUD.

## Conclusions

People with SUD go almost twice as often to the GP as comparators and more than seven times more often to the psychiatrist. They are also more than six times more frequently admitted to the hospital than comparators. People in treatment for alcohol use disorders make significantly more use of health services and health service providers than people in treatment for disorders related to street drugs. The use of health services remains quite stable in the five years before and after the moment people with SUD entered into treatment for their SUD.

## Data Availability

The full dataset of this study is not available due to restricted access to the IMA database.

## Abbreviations

TDI: Treatment Demand Indicator
IMA: InterMutualistisch Agentschap
SUD: Substance use disorder
GP: General practitioner
ED: The emergency departments
CI: Confidence interval
DALY: Disability-adjusted life-years
HIV: Human immunodeficiency virus
HCV: Hepatitis C Virus
MDMA: 3,4-Methyl enedioxy methamphetamine
NIN: National Identification Number
